# Did we describe what you meant? Findings and methodological discussion of an empirical validation study for a systematic review of reasons

**DOI:** 10.1186/1472-6939-15-69

**Published:** 2014-09-27

**Authors:** Marcel Mertz, Neema Sofaer, Daniel Strech

**Affiliations:** 1 Research Unit Ethics, Institute for History and Ethics of Medicine, University Hospital Cologne, Herderstr. 54, D-50925 Cologne, Germany; 2 Institute for History, Ethics and Philosophy of Medicine, Hannover Medical School, Carl-Neuberg-Str. 1, D-30625 Hannover, Germany; 3 Centre of Medical Law & Ethics, King’s College London, Strand, London WC2R 2LS, UK

**Keywords:** Systematic review of reason, Systematic review, Methodology, Medical ethics, Bioethics, Empirical ethics, Post-trial access

## Abstract

**Background:**

The systematic review of reasons is a new way to obtain comprehensive information about specific ethical topics. One such review was carried out for the question of why post-trial access to trial drugs should or need not be provided. The objective of this study was to empirically validate this review using an author check method. The article also reports on methodological challenges faced by our study.

**Methods:**

We emailed a questionnaire to the 64 corresponding authors of those papers that were assessed in the review of reasons on post-trial access. The questionnaire consisted of all quotations (“reason mentions”) that were identified by the review to represent a reason in a given author’s publication, together with a set of codings for the quotations. The authors were asked to rate the correctness of the codings.

**Results:**

We received 19 responses, from which only 13 were completed questionnaires. In total, 98 quotations and their related codes in the 13 questionnaires were checked by the addressees. For 77 quotations (79%), all codings were deemed correct, for 21 quotations (21%), some codings were deemed to need correction. Most corrections were minor and did not imply a complete misunderstanding of the citation.

**Conclusions:**

This first attempt to validate a review of reasons leads to four crucial methodological questions relevant to the future conduct of such validation studies: 1) How can a description of a reason be deemed incorrect? 2) Do the limited findings of this author check study enable us to determine whether the core results of the analysed SRR are valid? 3) Why did the majority of surveyed authors refrain from commenting on our understanding of their reasoning? 4) How can the method for validating reviews of reasons be improved?

## Background

### Rationale and an example of a systematic review of reasons

A systematic review of reasons (or arguments, or reflections on ethical issues; SRR for short) in philosophical bioethics [1-6] is a new way for decision-makers to obtain comprehensive information about specific ethical topics [7]. In such a review, the goal is not to normatively answer an ethical question (e.g. whether post-trial access to trial drugs is morally required or not) [8]. Its goal is rather to address the *empirical* question of which reasons (or arguments, or reflections) have been given in the academic literature when considering an ethical question, operationalized by identifying relevant quotations where reasons are given [7]. This is done in order to present detailed information on these reasons – whether they are for or against the practice under discussion; to which “type” of more general reason (e.g. benevolence, avoid exploitation, incentives…) they belong – and thus to provide decision-makers (e.g. guideline developers) with a comprehensive overview of the reasons discussed. Thus, this method is purely descriptive; nor does it review and synthesize quantitative data as reviews of clinical trials or survey research do [9,10].^a^ The rationale for using this method is to avoid bias when summarizing reasons that are published in scientific literature, and to provide a comprehensive set of reasons for or against a course of action, especially when developing policy or guidelines, as a biased or incomplete sample of reasons may lead to ethically problematic recommendations (see also [7,9]).

One such SRR was performed for the question of why post-trial access (PTA) to trial-drugs should or need not be provided [4]. This was also the first practical application of the SRR method, which analysed 75 references out of 2039 publications about PTA that were published between January 1991 and September 2009. The results of the review, as well as the actual method used, are of no concern here (for those, see [4,11]); rather, we shall ask whether this review – and, by implication, any other SRR – can be empirically validated, i.e., whether it is possible to verify the coding done by the reviewers in order to classify different kinds of reasons given in the references.

### The need for and obstacles to validation of a systematic review of reasons

Such verification primarily aims to check that the reviewers understood the author’s reasoning correctly; hence, the reviewer’s understanding of the reason is checked against the author’s understanding of this reason.

This proved to be harder than we first thought. By framing the validation process as checking the understanding of the reviewer against the understanding of the author(s), we introduced an intractable epistemological question: who is epistemically authoritative in deciding which understanding was “really” correct? Methodologically, neither reviewers nor author(s) can be awarded a clear-cut epistemic privilege in this matter. Even if there is such a thing as an epistemic “first person privilege” which could be attributed to the author(s), it does not extend to textual representations and meanings affected by intersubjective exchange. Or, speaking hermeneutically, it might even be wrong to suppose there is one “real” (“true”) understanding. In this case, the reviewer might also “override” the understanding of the author(s) – but on what grounds?

Of course, the solution of such general epistemological problems is beyond the scope of this article. We argue that the best one can pragmatically do is to try to minimize the potential for reviewer error, and to be critical when interpreting completed questionnaires, in order to allow interpretations in cases where there are sufficient reasons to think that it was the author who made an error, or where the author’s interpretation seems implausible to the reviewers.

Notwithstanding this difficulty validation is of great importance, because an SRR ultimately stands or falls with the correctness of its reason coding – even if it was able to minimize bias when selecting appropriate literature. Our trust in the results of an SRR should increase when it can be shown that the authors of the literature concur with the coding, and it should decrease if they do not.

One could argue that the main goal of an SRR is reached when a complete range of reasons has been coded with minimal bias, and that robust peer review is a sufficient check. We would reply that even expert reviewers are prone to subjective bias, and thus not able objectively to decide the correctness of the coding of extracted reasons. Therefore, validation of the coding is essential to an improved methodology of SRRs.

### Validation study

A pilot of such a validation was made in December 2011, followed by a full attempt in July 2012 by means of a survey with a questionnaire, addressed to the authors or author groups of the 75 references included in the review; some authors or author groups wrote more than one publication in the review. The idea was to *countercheck* the coding the review used by presenting this coding to the respective author(s) of a reference. The underlying hypothesis was that a counterchecking process would enable dialogical validation of the SRR for PTA, making further assessments of its quality possible. The survey tried to test this hypothesis as well as to explore the general possibility of validation of an SRR.

In the following, the method for this survey and its results are presented.^b^ Owing to the low response rate and the inherent methodological challenges we found in the course of this first validation study of reason-codings, our discussion focuses on these issues – not only in regard to our study, but also with a view to future validation studies of SRRs.

## Methods

This section describes the method used in conducting the validation study.

### “Reason mention” and “quotation”

In the original publication of the SRR on PTA, the term “reason mention” was used for an instance in a reference where a reason was identified. For the survey, the term “quotation” was introduced, as the authors or author groups surveyed had to check whether the coding (as a reason mention) of a certain *quotation* from their publication was correct (see below). The same quotation from a reference could be used more than once, i.e. considered to contain more than one reason. In this case, the quotation was included once with each coding in the questionnaire. Hence, we use “quotation” and “reason mention” interchangeably in our analysis of the survey data, below.

In the systematic review, quotations (reason mentions) were coded in four categories: broad reason type, narrow reason type, reason for/or against ensuring PTA; position taken by author. The questionnaire was based on the same coding.

### Questionnaire

**Figure 1:**
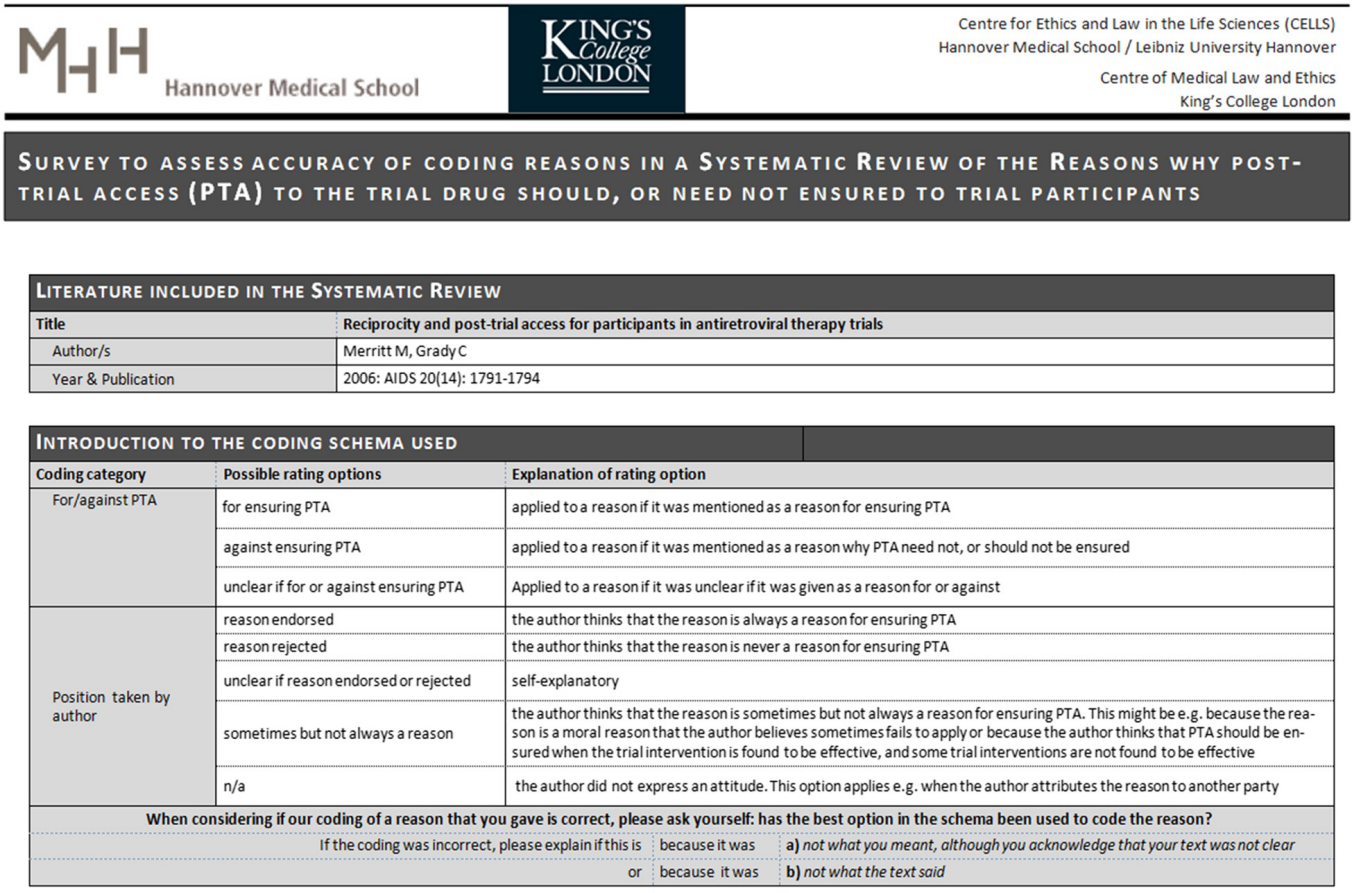
Example of the first page of the questionnaire.

**Figure 2:**
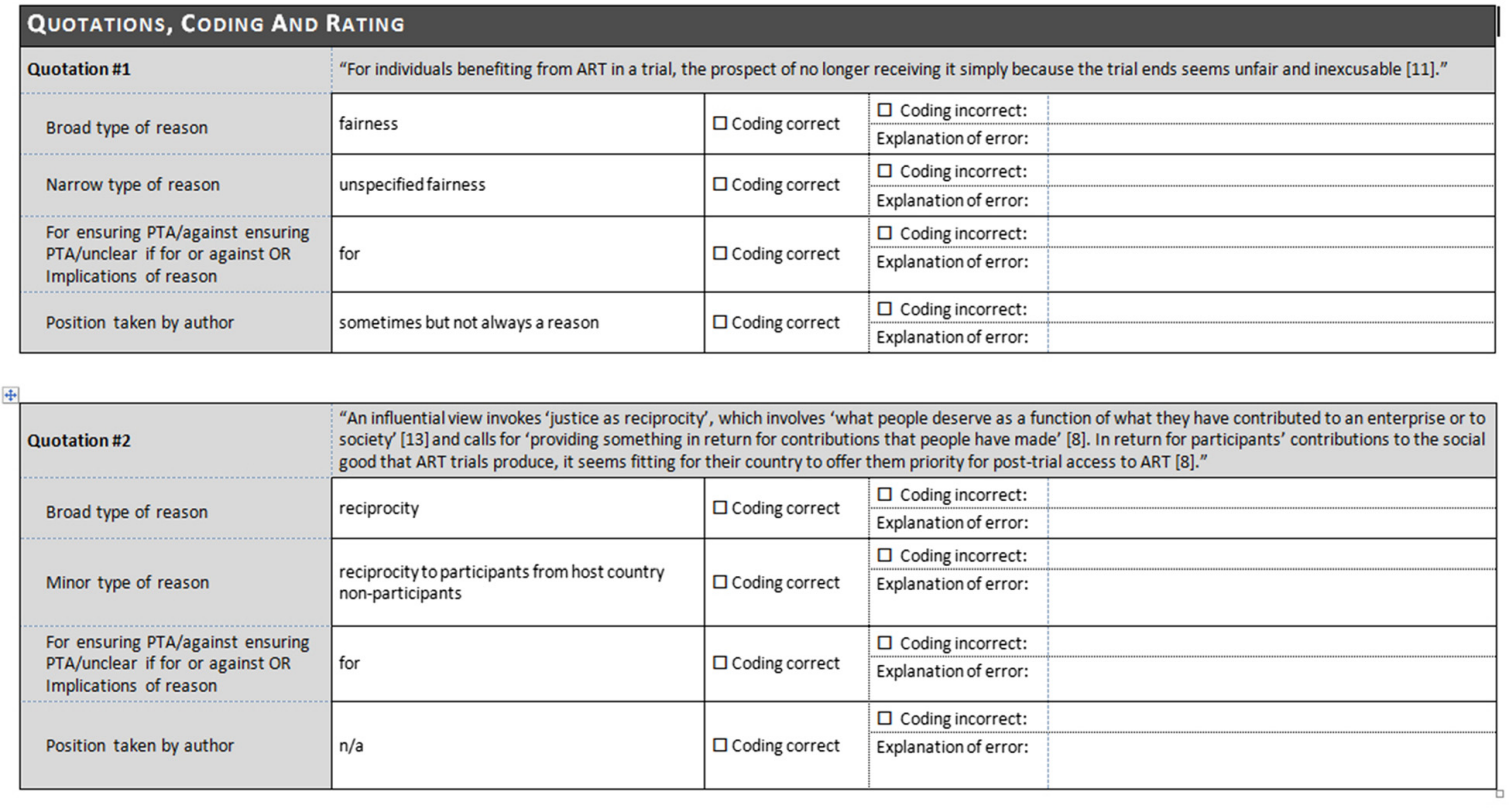
Example of quotations and coding.

**Figure 3:**
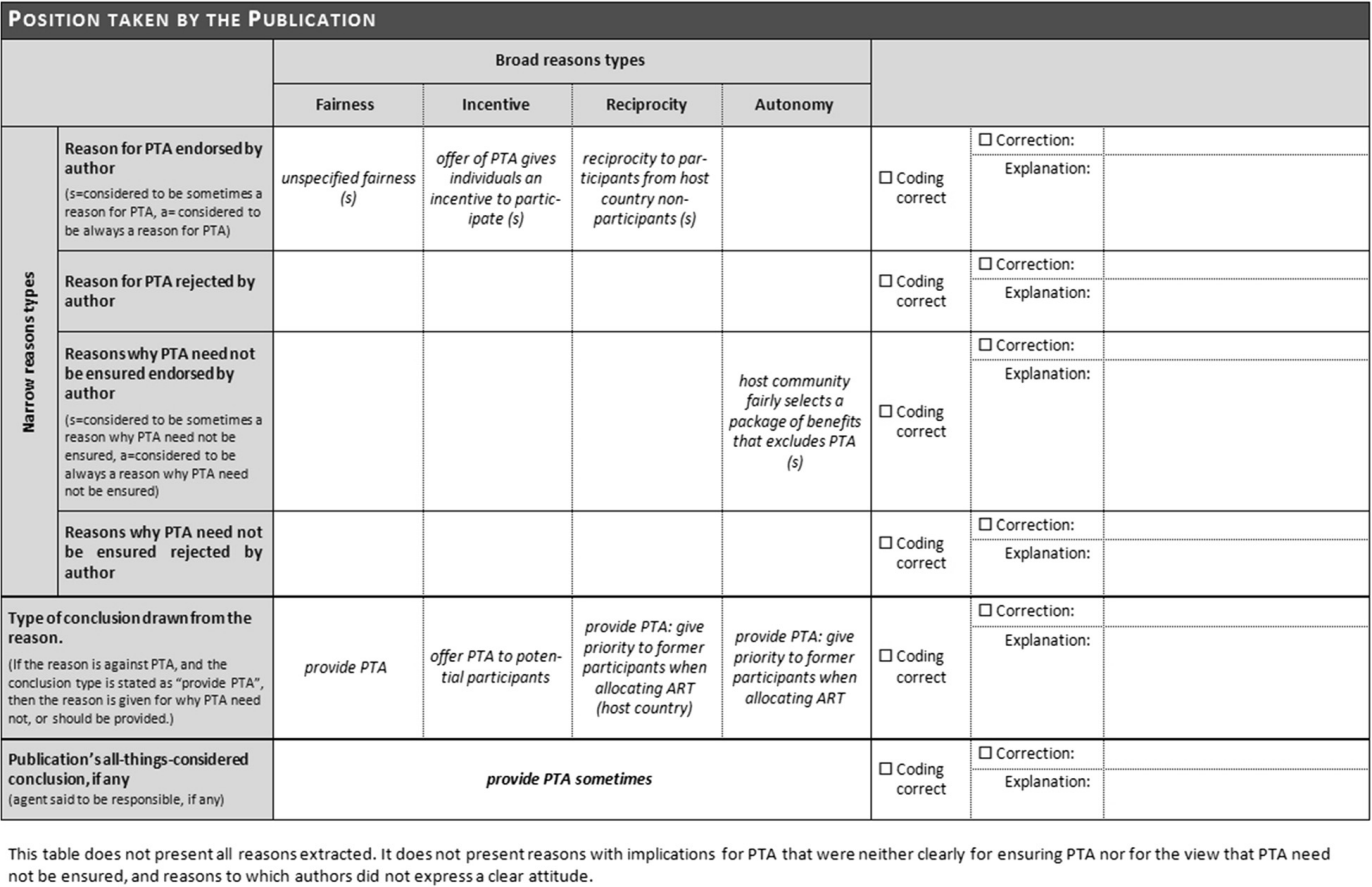
Example of table “Position taken by the publication”.

The survey comprised a questionnaire in Microsoft Word format that was e-mailed (with one reminder after 5 weeks) to the corresponding author of the reference (or to another author if no corresponding author could be identified or reached by e-mail). Each author received a separate, customized questionnaire with the quotations from her/his publication. An introductory text about the purpose of the survey and the background of the method of systematic review was included in the body of the e-mail. The questionnaire consisted of a front page with the publication data (title, authors etc.) of the publication in question, an overview of the coding used in the reviewing process, and an explanation of the rating options the addressees could apply to countercheck the coding (see Figure 1).Afterwards, each quotation that was identified as representing a reason in the addressee’s publication was displayed along with the corresponding coding (e.g. “avoid exploitation”, “reason for/against ensuring PTA”, “reason endorsed by the author” etc.). The addressees had the option to mark “Coding correct” or “Coding incorrect” – that is, whether they agreed with the coding or not – with the option to explain any error the reviewer made (see Figure 2).Finally, a chart showing the position taken by the publication was presented, again with the respective coding and “Publication’s all things considered conclusion, if any” (see Figure 3).

The last page of the questionnaire consisted of an “overall response” from the addressee (viz. author of the publication), chosen from “The coding was all correct”, “I corrected (some of) the coding and explained my corrections”, and “I refuse to comment on the quotations/coding”. Additionally, the addressees could make general remarks or give feedback on the coding schema.

### Ethics committee approval, informed consent and data handling

According to German regulation (Pharmaceutical and Medical Devices Laws, Medical Professional Law), no ethics approval is necessary for socio-empirical research that does not involve patients. Our study surveyed authors of bioethics papers. The handling of data safety and informed consent in this study was as following: Consent was considered to be implied when participants completed the online survey. No personal data of participants was collected in the survey. All data concerning the answers in the survey are stored on computers protected by the security policy of Hannover Medical School.

## Results

In this section, we present the results of the validation study.

### Response

Out of 75 references, 11 (15%) were excluded from the survey for various reasons (see Table 1). From the remaining 64 references (85%), 9 (12%) were used for a pilot survey that was sent to pre-selected authors. So, 55 references (73%) were part of the main survey. As there were no vital differences between the questionnaire used in pilot-testing and that used in the actual survey, the results of the pilot were added to those of the main survey (the only differences were details of the layout).

**Table 1:** Responses

Overall references in systematic review:	n = 75
Excluded references or questionnaires not sent:	11/75 (15%)
*No valid e-mail address found*	*2/75 (3%)*
Not transformed into questionnaire*	*4/75 (6%)*
Explicit rejection in pilot survey**	*3/75 (4%)*
No reaction in pilot survey**	*1/75 (1%)*
Unburden author who was already in pilot survey**	*1/75 (1%)*
Questionnaires sent (vs. overall references):	64/75 (85%)
*Pilot survey*	*9/75 (12%)*
*Main survey*	*55/75 (73%)*
Overall responses (vs. questionnaires sent):	19/64 (30%)
*Completed responses:*	*13/64 (20%)*
*Incompleted responses:*	*5/64 (8%)*
*Other responses:*	*1/64 (2%)*
No responses (vs. questionnaires sent):	45/64 (70%)
Adjusted response rate (only completed responses):	**13/64 (20%)**

*Due to possible conflict of interests with certain authors/author groups, or knowing beforehand that certain authors would be unable to participate.

**Relates only to authors/author groups from whom more than 1 reference was included in the review, and who had participated with 1 of their references in the pilot study (excluded references = total references by authors/author group minus the 1 reference in the pilot study).

The overall response rate (pilot survey included) was 30% (19 questionnaires). Completed responses comprised 20% (13 questionnaires) of all questionnaires sent (9 completed questionnaires and 4 reconstructed questionnaires^c^). Incomplete responses – declining to participate in the survey, e.g. due to limited time – were received to 8% (5 questionnaires) of all questionnaires sent; 2% (1 questionnaire) consisted only of remarks concerning the methodology of the survey. There were no responses from 70% (45 questionnaires) of all questionnaires sent. So, the adjusted response rate, i.e. only considering completed responses, was 20% (13 questionnaires) (See also Table 1).

### Quotation counts

Had it been possible to send questionnaires for all 75 references of the SRR, 781 quotations (or reason mentions) would have been included. Per questionnaire, the number of quotations would have been between 1 and 66, with an arithmetic mean of 10.41, a lower quartile of 3, a median of 6 and an upper quartile of 12–13; the mode would have been 2.

In the 64 questionnaires (representing the same number of references) actually sent, 709 quotations (91% of all quotations) were included in the total. The range was again from 1 to 66, but with an arithmetic mean of 10.07; quartiles and mode were identical to those reflecting all 75 references (3, 6, 12–13 and 2, respectively).

The 13 completed responses addressed 98 quotations in total, making up 13% of all quotations in the review and for 14% of those in the questionnaires sent. The range was from 1 to 34, with an arithmetic mean of 7.54, a lower quartile of 4, a median of 5, and an upper quartile of 8–9. The mode was 4 (See also Table 2).

**Table 2:** Quotations

Total quotations in systematic review (= in 75 references):	n_1_ = 781
*Quotations in one questionnaire:*	
*Range:*	1–66
*Arithmetic mean:*	10.41
*Quartiles (Q*_*1*_*, Q*_*2*_*/median, Q*_*3*_*):*	3, 6, 12–13
*Mode:*	2
Total quotations in questionnaires sent (= in 64 references):	n_2_ = 709 (91% of n_1_)
*Quotations in one questionnaire:*	
*Range:*	1–66
*Arithmetic mean:*	10.07
*Quartiles (Q*_*1*_*, Q*_*2*_*/median, Q*_*3*_*):*	3, 6, 12–13
*Mode:*	2
Quotations total in completed responses (= in 13 references):	n_3_ = 98 (13%/14% of n_1_/n_2_)
*Quotations in one questionnaire:*	
*Range:*	1–34
*Arithmetic mean:*	7.54
*Quartiles (Q*_*1*_*, Q*_*2*_*/median, Q*_*3*_*):*	4, 5, 8–9
*Mode:*	4

### Coding validation

Out of 13 completed responses (completed or reconstructed questionnaires), 4 questionnaires (31%) of the addressees marked that all coding in the questionnaire was correct, and 9 questionnaires (69%) that some coding in the questionnaire was incorrect. For the sake of simplicity, we did not differentiate between minor incorrectness – e.g. further specifications of the coded reason type – and major incorrectness – e.g. subsumption error or a misunderstanding of the citation (“not what the text said”).

This finding has to be contrasted with the proportion of quotations that were deemed incorrect. The overall sum of quotations coded (in 13 questionnaires) was, as seen above, 98. For 77 quotations (79%), all coding was deemed correct, for 21 quotations (21%), some coding was deemed to need correction.

The greatest number of errors was found in the coding ascribing the reason as being “for ensuring PTA” or “against ensuring PTA” (43%, i.e. 9 quotations of the 21 quotations with incorrect coding). One “neutral” author (MM) with a master’s degree in philosophy, who was also not involved in the initial coding or the publication of the review findings that form the basis of this survey, double-checked all 21 codings that were deemed incorrect by the original authors. In 14 cases he verified that the initial coding was somewhat wrong. In 11 cases the survey respondents indicated that they endorsed a reason (for or against PTA) or took a position while the initial coding said they did not, or *vice versa*. In another 3 cases he verified claims of incorrectness that dealt with subsuming the reason under the correct reason type (for example, subsuming the reason under “legality”, but no legislation has been mentioned in the citation, or explicit denial from the authors that they think that “charity”, the coded broad and narrow reason type, is a rationale for PTA in the discussed context). In 6 cases he could not verify the claim of incorrectness in the initial coding. In 1 case he decided that the remark of the author is an explanation in order to make the original citation more understandable, rather than a correction; this result was added to the number of non-verified incorrect initial codings. Accepting this counter-countercheck, only 14% of all quotations were wrongly coded.

Some survey respondents explained why our coding was incorrect using the space given in the survey instrument. For example, some highlighted that the specific reason should be relabelled to “psychological harm” instead of “psychological health” or “well-being” instead of “health”. Other added minor specifications such as “avoid exploiting the *poor* of the country” instead of “avoid exploiting the host country” or that “cost of PTA” is just one of the factors for providing PTA.

The “Position taken by publication” chart was claimed to be wrong in 31% (4 questionnaires) of the 13 questionnaires; in 61% (8 questionnaires), it was claimed to be correct. In 8% (1 questionnaire), the chart was not completed by the respondents. The neutral author (MM) could not verify 2 claims of incorrectness. In the other 2 cases he was unsure whether the survey respondents claimed incorrectness or whether they were rather trying to explain their point of view in more depth. Again, accepting this counter-countercheck, only 15% of the questionnaire charts were actually wrongly coded (See also Table 3).

**Table 3:** Results of coding validation

Questionnaires with completed response:	n = 13
All coding in questionnaire correct:	4/13 (31%)
Some coding in questionnaire incorrect:	9/13 (69%)
“Position taken by publication” chart coding correct:	8/13 (61%)
“Position taken by publication” chart coding incorrect*:	4/13 (31%)
“Position taken by publication” chart not filled out:	1/13 (8%)
Overall sum of quotations coded (in 13 questionnaires):	n = 98
*Quotations coding correct*	*77/98* (79%)
Quotations coding incorrect**	*21/98* (21%)
*Coding corrected by author (vs. sum coding incorrect):*	*19/21* (90%)
*Coding not corrected by author (vs. sum coding incorrect):*	*2/21* (10%)
Quotations coding incorrect: Type*** (vs. sum coding incorrect):	
*Broad type of reason:*	*5/21 (24%)*
*Narrow type of reason:*	*5/21 (24%)*
*For ensuring PTA/against ensuring PTA:*	*9/21 (42%)*
*Position taken by author:*	*2/21 (10%)*
Countercheck “Position taken by publication” (vs. quotations):	
*Verified correctness of “Position taken by publication” coding:*	*10/13 (77%)*
*Verified incorrectness of “Position taken by publication” coding:*	*2/13 (15%)*
*(“Position taken by publication” chart not filled out):*	1/13 (8%)
Countercheck coding:	
*Quotations coding correct (vs. overall sum of quotations):*	*84/98* (86%)
*Quotations coding incorrect (vs. overall sum of quotations):*	*14/98* (14%)
*Verified incorrectness of coding (vs. sum coding incorrect):*	*14/21 (67%)*
*Broad type of reason*	*2/21 (10%)*
*Narrow type of reason*	*1/21 (5%)*
*For ensuring PTA/against ensuring PTA*	*9/21 (43%)*
*Position taken by author*	*2/21 (10%)*
Verified correctness of coding**** (vs. sum coding incorrect):	*7/21 (33%)*

*Taken as incorrect when at least one coding in the chart was deemed incorrect.

**Taken as incorrect when at least one coding concerning the quotation was deemed incorrect.

***Multiple answers were possible, means that more than one type could have been wrong.

****One case was an explanation rather than a correction; it was added to this category.

## Discussion

### Validation study and open methodological questions

The results above present the findings of a first attempt to validate the core results of an SRR (published elsewhere) by means of a structured author check. Due to the low response rate of 20% (n = 13) we received feedback on only 13% (98) of the 781 original quotations in the 75 references that we identified as text that describes one or several reasons in the systematic review. This response rate corresponds to only 21% of all authors or author groups whose papers were analysed in the SRR (n = 63).^d^ More importantly, it corresponds to only 14% of the total 709 quotations in the sent questionnaires.

Of these 98 quotations the original authors said that 77 (79%) were coded correctly. The low response rate, the validation of only a subset of the quotations and the fact that 21 (21%) of our codings for quotations were described as being somewhat incorrect raises at least four open questions that are relevant not only to the particular review of reasons assessed in this study, but also to further similar studies:

1) What are the reasons for a coding to be deemed incorrect?

2) All things considered, do the limited findings of this author check study permit any conclusions to be drawn as to whether the core results of the analysed SRR are valid or invalid?

3) Why did 70% (n = 45) of surveyed authors of argument-based literature refrain from validating the results of an external (re-)analysis of their argumentation and underlying reasons, published in an established bioethics journal (despite two requests to send even the shortest feedback or to comment on the reasons for the unwillingness to respond)?

4) Is there a better methodological approach to validate the results of an SRR than the one we used in this study?

Questions 1) and 2) are crucial to the particular review of reasons assessed in the validation study. All questions are relevant to further similar studies, however, and for that reason, we want to focus on these aspects in the following subsections.

### Reasons for a coding to be deemed incorrect

As the validation process checks the understanding of the reviewer (= coding) against the understanding of the author (= quotation), there are two overriding groups of sources for a coding to be deemed incorrect: methodological or hermeneutical failures/hindrances of the reviewers, and failures/hindrances of the author(s).

Regarding the former group, there are three main issues accounting for coding that was deemed incorrect by a participant, starting with methodologically more sensitive issues and ending with a more technical one: (i) potential interpretation errors (the coder misunderstood the content of the read paper); (ii) potential subsumption errors (the coder subsumed a reason mention under the wrong code); (iii) transcription errors (the coding was wrongly transcribed from the data base into the questionnaire).

Regarding the latter group, participants, i.e. the authors of the cited papers, may have failed to determine in a veridical way if the coding was correct, for example because of (i) carelessness when answering the survey, (ii) misunderstandings concerning the coding system, or (iii) for the simple reason that the participants did not (re-)read their papers and re-familiarise themselves with what their papers actually say.

A further sources of failure “between” the two above-mentioned may also be considered: potential differences in the way a reason is coded that lead to differences in how the correctness of the coding is assessed (e.g., compromises in the process of coding were unavoidable in order to keep the number of codes manageable, but authors might have other criteria for coding their reasons, and thus have rated the coding given as “incorrect”).

To avoid such errors, further measures to ensure the validity of the survey’s results would be needed, such as involving neutral third parties to double-check cases of conflicting codings/ratings. From a more hermeneutical point of view (see also Background section), and in line with some strands in qualitative research methodology [12,13], one could also try to engage in a dialogue with the author when codings/ratings conflict in order to find a coding/rating both can agree with after exchanging their understanding, reasons and motives. Theoretically, this will probably imply accepting that understanding is always of dialogical nature, and therefore can never be pinpointed to an empirically “simple” objective measure, such as a tick box in a survey – but that would also lead to the question of whether the survey method is the most appropriate to validate an SRR.

### Validity of the analysed SRR

All things considered, we conclude that our attempt to validate the core results of an SRR was at most a partial success, as only 13% of all quotations of the review and 14% of the quotations in the sent questionnaires were covered by the response, reducing representivity. Nevertheless, the fact that 79% of the quotation codings and 61% of the “position taken by publication” chart codings were rated as “correct” indicates a positive evaluation and therefore at least a partial validation of the core results of our review of reasons. Where the coding was rated as “incorrect”, it could not be verified in all cases by a neutral author, leading to 86% and 77%, respectively, of correct coding when accepting this counter-counterchecking. Also, most corrections were only minor (specifications of the coded reason type) and did not amount to subsumption errors or to a complete misunderstanding of the citation; the only exception was some “reason endorsed/not endorsed” coding.

However, the authors of the reviewed literature that positively responded concluded that 21% of our reason codings were “incorrect”. In particular, 42% of “for/against ensuring PTA” and 10% of the “Position taken by author” codings were deemed wrong. These two facts highlight the need to improve the validity and reliability of such coding – on the researcher’s side as well as on the participant’s side.

### Reasons for non-response to the validation survey

There could be several potential reasons for the low response rate in this survey: the amount of time needed to answer the questionnaire; possible misunderstandings of the purpose of the survey; possible incomprehensibility or complexity of the questionnaire; the time passed between publication (e.g. 1991) and the survey asking about the publication (which could also mean that authors have changed their position without noticing, or without acknowledging that their once published argument was difficult to maintain against the backdrop of their current position); that authors did not understand the methodological relevance of systematic reviews of reasons and their validation, or that authors explicitly rejected the approach of an SRR as a way to engage with their arguments and/or as a way of handling conceptual/normative issues.

We performed a non-responder analysis to understand the main barriers to participation in our study. However, only 6 authors or author groups (10% of the 63 authors/author groups) explained why they were unwilling to check our attempt to code their reasons for or against ensuring PTA to trial participants. The reasons of these 6 authors or author groups were: unfamiliarity with the methodology used (n = 2); being critical of the methodology of an SRR in general; not being the author of the paper; being a journalist and not a scientist (having written a news article for a journal, but not a scientific paper); not having enough time to answer the survey. The 4 authors that responded to the pilot survey did not raise major concerns about the comprehensibility of the survey material (though one author mentioned “unfamiliarity with the methodology used” and refused to participate in the survey for this reason). If problems in the understanding of our survey were a major barrier to participation we would have expected at least some surveyed authors to have asked for further explanation of our survey material (only 2 authors asked specific questions concerning the coding of one or two quotations).

While not having enough time might be an important and common reason for not participating, as is the fact that a paper was written long time ago, another specific possible explanation should be mentioned. Perhaps a certain lack of understanding of the idea of an SRR, its relevance to practice-oriented bioethics and the methodological necessity of counterchecking the coding used, even some kind of prejudice against “empirical ethics” in general, was a reason for not even responding in a negative way (refusal). If this was a significant reason – which we cannot know for sure based on our data – it should be acknowledged as a pressing problem for the method of systematic reviews of reasons, as this could not be amended just by improving the survey method for validation (e.g. reducing the complexity of the questionnaire, providing more assistance in filling out the questionnaire, or performing the survey in a completely different way). We therefore argue that it could be worthwhile investing more time and effort in demonstrating the value of scientific attempts to assess whether others are able to understand what an author of argument-based literature has written – if only as a possible way to further motivation for participation in a validation study.

This investment is, we think, eminently worthwhile in the interdisciplinary field of bioethics, which would profit from such attempts to assess the external comprehensibility, the validity, and the codability of argument-based literature. After all, it should be of interest to the authors of argument-based literature whether their reasons have been correctly understood, especially when their literature has been included into a systematic review in order to (better) inform decision-makers. For example, as we were told by one of the organizers of the new revision of the Declaration of Helsinki, the results of the SRR that we tried to validate in this survey were circulated among the invited experts who contributed to the drafting of new and modified content on the PTA issue (though of course we do not know if the systematic review actually had an impact on the revision).

### Methodological approaches to validate an SRR

We received little feedback highlighting any sort of methodological shortcomings in our survey. One might criticise the length of some survey forms owing to the number of quotations identified in certain papers (up to 66), so that our personalized survey form reached 23 pages in some extreme cases. However, as Table 2 shows, it has to be considered that 25% of the questionnaires consisted of fewer than 3 quotations (lower quartile), and 50% had fewer than 6 quotations (median), respectively; even 75% (lower three quartiles) contained fewer than 13 quotations. Most often, the questionnaires consisted of 2 quotations (mode). Also, some authors of papers with many quotations participated in our study and did not raise any concerns about the length of the survey. One might also argue that even when the survey form was long, it should be easy for an author to understand the original quotations from her/his own paper. While some longer quotations increased the length of the survey form, the coding of such quotations that we asked the authors to check for validity consisted only of single words. Furthermore, we used the same pattern to structure the survey form as in the original publication of our review findings, which was positively reviewed during external peer review for the journal *Public Health Ethics*[4]. Since its publication in 2011 this paper has been cited by 5 other papers (excluding citations by new papers from the authors of this systematic review), none of which raised concerns regarding the presentation of quotations. But even taking into account all the above-mentioned actual and possible limitations and sources of error (see also Table 4), we have good reasons to believe that our methodology for validating the coding of reasons as core results of a systematic review of argument-based literature was appropriate and can be recommended for future similar efforts: (i) generally, a survey is a “tried and tested” social science research methodology, and “author check” methods are established in qualitative research (even though two survey participants stated unfamiliarity with the methodology used); (ii) there seems to be no viable alternative to sending individual questionnaires (to each author or author group of a paper) to validate the coding of an SRR (this cannot be done by one questionnaire for all participants); (iii) as the participants were invited not only to tick a box when they deemed a coding incorrect, but to clarify why the coding was wrong, it was possible for the reviewers to interpret – to a certain degree – the understanding of the reasons by the participants; this made “overriding” participant’s evaluation by reviewer’s understanding feasible, when the interpretation seemed too implausible to them (e.g. not plausible on logical or hermeneutical grounds against the backdrop of the argumentation given in the paper).

**Table 4:** Actual/possible limitations & error sources

Very low response rate due to …	… amount of time needed to answer questionnaire
	… misunderstandings of the purpose of the survey
	… possible incomprehensibility or complexity of the questionnaires
	… time passed between publication and survey
	… the changing of the position of the author(s) towards PTA
	… author(s) do not understand the methodological relevance of SRRs
	… authors explicitly rejecting approach of SRR
Coding validation errors/hindrances on behalf of the reviewer(s), due to …	… potential interpretation errors (coder misunderstood content of read paper)
	… potential subsumption errors (coder subsumed a reason mention under wrong code)
	… potential transcription errors (coding wrongly transcribed from data base)
Coding validation errors/hindrances on behalf of the author(s)/participant(s), due to …	… carelessness when answering the survey
	… misunderstandings concerning the coding system
	… not having (re-)read the paper, and not having re-familiarised oneself with what the paper actually say
Coding validation errors/hindrances on behalf of the reviewer(s) and the author(s)/ participant(s), due to …	… potential differences in the way a reason is coded by reviewers and by authors
	… different understanding of the reason (and its implication relevant for coding)

Nevertheless, we highly recommend intensive pre-testing of the willingness of authors of argument-based literature to participate in such a validation study. In our study we were probably too optimistic in this regard. Furthermore, we do not imply that other methods might not be more successful, or more appropriate than ours; indeed, we recommend that other methods for validating results of systematic or narrative reviews of argument based literature be tried, e.g. conducting a parallel review – or at least analysis of the found literature – by another, independent research group (providing validity by investigator triangulation), assessing and comparing (other) narrative reviews (providing validity by data triangulation), using focus group approaches with selected experts in the field (providing expert validity), or trying to establish a (limited) dialogue with the participants concerning their estimates of accurateness of the coding (providing validity through dialogical/communicative validation).

## Conclusions

Our survey demonstrates that empirical research methods in bioethics are useful not only for retrieving “facts” about the topic at hand (in our systematic review: the range or reasons given in the scientific literature), but also as a means to evaluate methodological quality and to increase the validity of these “facts”. This paper further illustrates the challenges and opportunities of the validation approach (author check survey) reported. In doing so, it also demonstrates the importance of validation procedures for the coding used in an SRR. Even though the response rate was low, this validation study gave some validity to the SRR about PTA, and triggered methodological reflection about such validation studies in general. Further means of providing evaluation and validation of SRRs should be sought.

## Endnotes

^a^Another approach to systematic reviews in philosophical bioethics or “argument-based medical ethics”, which might be called a “systematic review of conclusions” [7] was proposed by McCullough et al [1,8]. This approach aims to answer an ethical question normatively by reviewing and especially appraising the all-things-considered conclusions of a body of literature.

^b^The dataset on which the survey was based was created by two authors (NS, DS), of whom one (NS) had the initial idea for creating the dataset, and took a leading role in analysing and extracting data.

^c^These questionnaires were reconstructed from an e-mail response (the authors did not return a completed questionnaire, but summarized her/his answers in an e-mail).

^d^As 8 authors or author groups had more than 1 publication in the systematic review, the count of authors or author groups does not sum to 75 (the count of references in the review), but only to 63.

## Abbreviations

PTA: Post-trial access; SRR: Systematic review of reason.

## Competing interests

Financial competing interest: The authors declare that they have no competing interests.

Non-financial competing interest: The authors report findings of a validation study concerning a new methodology for systematically reviewing and coding reasons in argument-based literature that has been first described and applied by two authors of this paper (NS and DS). The results of the first application of this methodology provided the content of this validation study.

## Authors’ contributions

MM and DS designed the study and wrote the first draft of the paper. NS contributed to the study design and final draft. The dataset on which the survey was based was created by NS and DS, of whom NS had the initial idea for creating the dataset, and took a leading role in analysing and extracting data. MM acted as “neutral” author as well. All authors read and approved the final manuscript.

## Pre-publication history

The pre-publication history for this paper can be accessed here:

http://www.biomedcentral.com/1472-6939/15/69/prepub
